# Femoral rotational osteotomy for femoroacetabular impingement: A systematic review

**DOI:** 10.1016/j.jor.2023.12.015

**Published:** 2023-12-22

**Authors:** Chase T. Nelson, Charles R. Reiter, Matthew Harris, Carl Edge, James Satalich, Conor O'Neill, John Cyrus, Alexander Vap

**Affiliations:** a Virginia Commonwealth University School of Medicine, Virginia Commonwealth University, VCU Medical Center, 1201 E Marshall St #4-100, Richmond, VA, 23298, USA; b Department of Orthopaedic Surgery, Virginia Commonwealth University Hospital, Box 980153, Richmond, VA, 23298-0153, USA; c Department of Orthopaedic Surgery, Duke Health, 200 Trent Dr Ste 1F, Durham, NC, 27710, USA; d Health Sciences Library, MCV Campus at Virginia Commonwealth University, 509 N. 12th St., Box 980582, Richmond, VA, 23298-0582, USA

**Keywords:** Femoroacetabular impingement, Femoral rotational osteotomy, Femoral version, Systematic review

## Abstract

**Purpose:**

To synthesize existing literature regarding the indications and outcomes of femoral rotational osteotomies (FDO) for femoroacetabular impingement (FAI) due to.

**Methods:**

Medline, Cochrane, and Embase were searched using keywords “femoroacetabular impingement”, “rotational osteotomy” and others to identify FAI patients undergoing FDO. Double-screened studies were reviewed by blinded authors according to inclusion criteria. Data from full texts was extracted including study type, number of patients, sex, mean age, surgical indication, type of dysplasia, associated pathology, surgical technique, follow-up, and pre-op/post-op evaluations of the following: impingement test, femoral version (FV), ‘other angles measured’, outcome scores, range of motion (ROM).

**Results:**

7 studies including 91 patients (97 FDO surgeries), 73 females (80 %) with mean age of 28.3 years, and follow-up mean of 2.44 ± 2.83 years. Pain or impingement was the most common clinical indication, while others included aberrant FV and ROM measurements for both anteverted and retroverted femurs. There were reports of FDO being performed with concomitant procedures addressing other pathology. Various outcome scores and ROM measurements showed postoperative improvement after FDO. Complication data was sparse, preventing aggregation. The rate of unplanned reoperation was 40 % (where reported), with ‘hardware removal’ being the most common.

**Conclusions:**

FDO is effective in treating FAI due to increased FV, improving clinical symptoms, and potentially delaying articular degeneration. Hardware removal surgery remains an inherent risk in undergoing FDO. Further work is needed to discover indications warranting FDO as a primary treatment versus hip arthroscopy.

**Level of evidence:**

This review contains 4 studies with Level IV evidence and 3 studies with Level III evidence.

## Highlights

• Femoral Rotational Osteotomies safe and reliable treatments for impingement in hips with femoral version.
• Femoral Rotational Osteotomies may delay progression of osteoarthritis.
• Future studies may evaluate the impact of high rates of reoperation for hardware removal.

## 1 Introduction

Femoroacetabular impingement (FAI) has multiple etiologies including congenital dysplasia of the acetabulum or femur, post-traumatic deformity, or being secondary to conditions like slipped capital femoral epiphysis (SCFE) and osteonecrosis.^1^, ^2^, ^3^ Various surgical strategies are employed to address the underlying pathology causing FAI with prior systematic reviews (SR) investigating surgical management of acetabular dysplasia and SCFE currently existing in the literature.^4^, ^5^, ^6^, ^7^, ^8^ FAI due to dysplastic femoral etiology can be treated both conservatively and with minimally-invasive hip arthroscopies, while femoral rotational/de-rotational osteotomies (FDO) have traditionally been reserved for definitive management in cases of increased or decreased femoral version (FV), or refractory to failed conservative management or arthroscopy.^1^^,^^9^^,^^10^ FDOs are well-established procedures utilizing a transverse cut along a strategic location on the proximal femur, allowing for rotation and repositioning of the femoral head within the acetabulum and secure fixation with either intramedullary nail, or an angle blade plate.^11^^,^^12^ Patients undergoing FDOs usually present with extremes in femoral version (FV) or Coxa Valga on imaging, and with pain or impingement, limited range of motion, gait disturbances, and accelerated wear to the articular structures of the hip.^2^^,^^13^ Likewise, FDOs have benefitted from improved techniques featuring more minimally invasive exposures that require fewer myotomies.^14^^,^^15^

The purpose of this systematic review is to benefit the literature by synthesizing data from studies and commenting on the state of FDOs in FAI management, as well as elucidating areas that may warrant future investigation and/or guide clinical decision-making.

## 2 Methods

### 2.1 Database search and registration

**Fig. 1:**
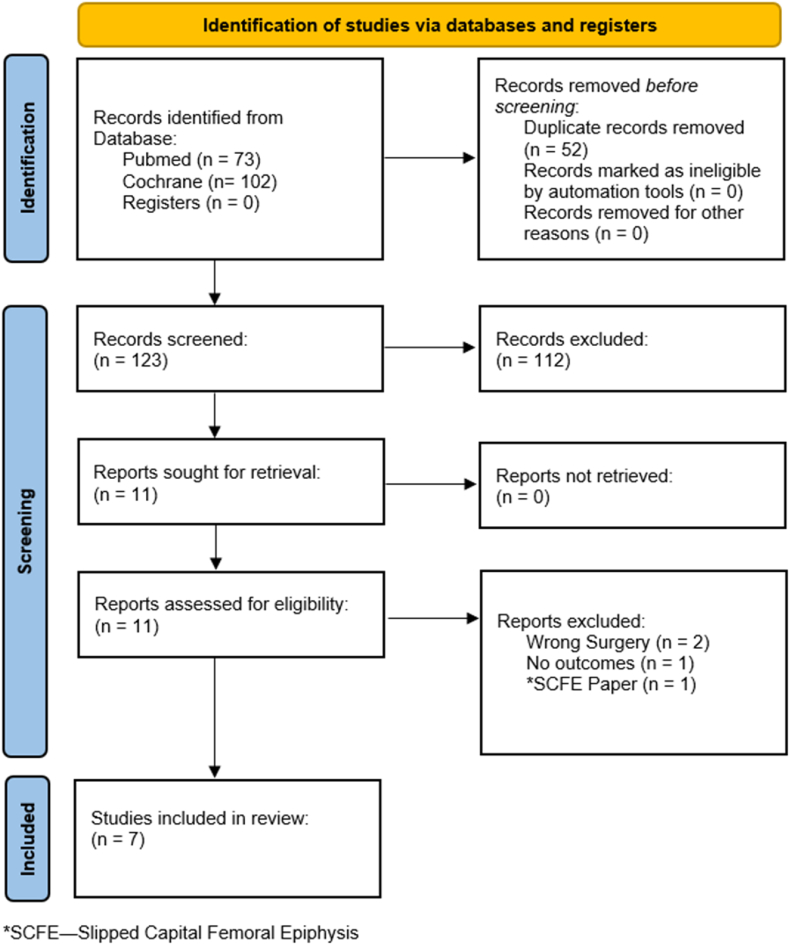
Details of database search as reported according to PRISMA guidelines.

A systematic search of Medline (Ovid), Cochrane, and EMBASE was conducted from inception of the database to February 2nd, 2023, by a research librarian using keywords and mesh terms (Appendices 1,2, and 3). Keywords and concepts included ‘femoral osteotomy’, derotation/rotation osteotomy, femoroacetabular impingement or FAI and ‘dysplastic hip impingement’. References were imported into Endnote reference management software (Clarivate, https://endnote.com/) and duplicates were removed (Fig. 1). Remaining references were exported and uploaded for screening according to eligibility criteria through Rayyan (https://www.rayyan.ai/). Prospective registration of the protocol for this systematic review was performed through PROSPERO (https://www.crd.york.ac.uk/prospero/display_record.php?ID=CRD42023401804).

### 2.2 Eligibility criteria and study selection

Abstracts and titles were screened first (Fig. 1). Two blinded reviewers (CN, CR) included studies in English that reported on FAI managed by rotational/derotational osteotomies. Case reports, case series, retrospective cohort studies, and randomized controlled trials were included. Studies were excluded if they did not directly investigate rotational/derotational osteotomies or if SCFE was the etiology of FAI. Likewise, reviews, non-human studies, cadaveric studies, and studies not published in English were excluded. A second full text review was performed on the remaining papers based on the same criteria to exclude any additional studies. A senior author (JS) resolved any conflicts between reviewers if indicated.

### 2.3 Data extraction

Two authors (CN, CR) independently extracted data points. The opposite reviewer then performed a second review, with any discrepancies being resolved through a senior author (JS). Data points for included the following: study type, level of evidence, number of patients, sex, mean age, surgical indication, type of dysplasia, associated pathology, type of osteotomy, surgical technique, follow-up duration, recurrence of impingement, and pre-op/post-op evaluations of the following: impingement test, femoral version, other angles measured, outcome scores, range of motion (ROM). Other information extracted included post-op complications, reoperations, ‘other outcomes’, and any salient findings from the studies.

### 2.4 Methodological quality assessment/risk of bias

JBI protocol checklist (https://jbi.global/critical-appraisal-tools) for cohort studies, case series, and case reports was used to evaluate methodological quality of included studies to analyze validity of methods, appropriateness of analysis, and quality of presentation for their given study type.

### 2.5 Statistical analysis

Meta-analysis was not performed. Categorical variables were summated and presented as counts or proportions, while continuous variables will be presented as means. Outcomes of interest were aggregated and reported in tables highlighting individual studies and their findings. The research team reported on findings from data synthesis as tables and figures.

## 3 Results

### 3.1 Literature search

Search of databases yielded 123 papers after duplicates were removed (Fig. 1). After screening of title and abstracts, 11 studies were included for full data extraction. After full text screening, 4 more were excluded resulting in 7 studies included in this analysis. Results were reported according to the PRISMA (Preferred Reporting Items for Systematic Reviews and Meta-Analyses) reporting standards (PRISMA).^16^

### 3.2 Data extraction and analysis

This study reviews 4 case reports/case series studies with level IV evidence, and 3 retrospective cohort studies with level III evidence.

### 3.3 Demographics

91 patients underwent 97 FDO surgeries, with 73 females (80 %) with a mean age of 28.3 years calculated by weighted means from reported study data. Follow-up intervals ranged from 1 year to 9.4 years with a weighted mean of 2.44 years (±2.83).

### 3.4 Indications for treatment

Six of 7 studies reported indications for surgery,^17^, ^18^, ^19^, ^20^, ^21^, ^22^ and pain and reduced ROM were the most cited indications (4 out of 7 studies). Details of decreased ROM were a function of increased or decreased FV and are reported in detail in Table 1. Likewise, impingement/FAI and a positive FABER and/or FADIR test were each cited in 2 studies.

**Table 1:** Included study details, patient demographics, surgical indication, type of dysplasia, associated path., and surgery.

Study	Study Design (Level of Evidence)	No. of Patients	No. Female	Mean Age	Surgical Indication	Type of Dysplasia	Associated Pathologies	Surgery Performed
**Cho (2021)**	Case Report (IV)	1	0	37	Hip pain limited ROM residual intra-articular FAI extra-articular impingement d/t femoral retroversion and high CCD angle (i.e., Coxa Valga)	Coxa Valga Femoral Retroversion	Linear/displaced intertrochanteric fracture 3yrs prior, treated with internal fixation.	Femoral Subtrochanteric Varus De-Rotation Osteotomy + Surgical Hip Dislocation
**LErch (2022)**	Retrospective Cohort (III)	20	17	28.6	–	Increased FV (n = 14) Decreased FV (n = 6)	–	Isolated Subtrochanteric Femoral De-Rotation Osteotomy
**Lerch (2022)**	Case Series (IV)	23 (25 hips)	22	26	Posterior/posterolateral hip pain Restricted ER in extension + posterior impingement test + FABER test Failed nonsurgical management Symptoms >6 months	Abnormally high femoral version	6 patients s/p prior surgery: 1 SHD for FAI 1 triple osteotomy as child 4 hip arthroscopy + labral resection*. *(2 patients underwent prior procedure twice, 1 suffered postop dislocation)	Femoral De-Rotation Osteotomy (avg. 20° of de-Rotation performed)* *Concomitant Procedures: - Surg. hip dislocation (n = 24) - CAM resection (n = 21) - Labrum Refixation (n = 9) - Acetabular rim trimming (n = 4) - Acetabular cartilage treatment (n = 2) - Fem. Cartilage treatment (n = 2) - Distalization of Great. Troch. (n = 1)
**Mastel (2020)**	Retrospective Analysis (III)	29 (33 hips)	24	29	Impingement Reduced internal rotation	Decreased anteversion	Articular degeneration: 7/33 Labral pathology: 25/33	Femoral De-Rotation Osteotomy
**Mastel (2022)**	Retrospective Matched Cohort (III)	10	6	33	Reduced internal rotation + FADIR test	Retroversion	Articular degeneration: 4/10 Labral pathology: 8/10	Femoral De-Rotation Osteotomy
**Tamaki (2020)**	Case Report (IV)	1	0	16	FAI due to retroversion	Retroversion	Redness of anterosuperior labrum Injured posterior acetabular cartilage	Subtrochanteric osteotomy and internal rotation (arthroscopic then open)
**Tanaka (2019)**	Case Series (IV)	7	4	25.9	**OA Patients**: TRO indicated when 1- No other viable joint preserving procedures 2- Joint congruency improved/widened joint space in Lauenstein's position on lateral radiographs. **ON Patient**: TRO indicated when >1/3 of Femoral head articular surface was intact on lateral radiographs (i.e., enough healthy articular tissue for repair to be efficacious)	Anteversion or retroversion. (Determined by location of OA on femoral head or acetabulum)	**6 OA patients**: 2 with pigmented villonodular synovitis, 1 s/p rotational acetabular osteotomy, 1 s/p pyogenic arthritis, 1 FAI 1 acetabular dysplasia **1 ON Patien**t: 1 s/p femoral neck fracture w/severe displacement *4 SCFE excluded	Transtrochanteric Rotational Osteotomy (TRO)

FAI—Femoroacetabular Impingement; S/p—status post; ROM—Range of motion; d/t—Due to; CCD—caput-diaphyseal angle; FV—Femoral Version; ER—External Rotation; FDO—Femoral Derotational Osteotomy; OA—Osteoarthritis; ON—Osteonecrosis; TRO—Transtrochanteric rotational osteotomy; OA—osteoarthritis, SCFE—Slipped Capital Femoral Epihphysis; FADIR—Flexion, adduction, internal rotation test; TRO—Transtrochanteric Rotational Osteotomy.

### 3.5 Types of dysplasia

All 7 studies^17^, ^18^, ^19^, ^20^, ^21^, ^22^, ^23^ commented on dysplasia pattern with femoral version (FV) being present. Cho et al.^22^ was the only study to list an additional indication (Coxa Valga).

### 3.6 Associated pathologies

Associated pathologies were reported in 6 out of 7 studies,^17^, ^18^, ^19^, ^20^, ^21^, ^22^ labral pathology was most common and present in 34/44 cases (77.3 %) across 3 studies.^17^, ^18^, ^19^ Articular degeneration was noted in 12/44 cases (27.3 %) across the same studies.^17^, ^18^, ^19^ There were 12 patients across 3 studies^20^, ^21^, ^22^ (n = 33 patients) who had received previous hip surgery on the affected joint prior to undergoing FDO operation. Prior surgeries included: 1 surgical hip dislocation (SHD), 1 pediatric triple osteotomy, 4 hip arthroscopies (2 undergoing procedure twice, 1 with postop dislocation), 1 internal fixation of intertrochanteric fracture 3 years prior, and 1 internal fixation of femoral neck fracture. The percentage of prior surgery among these studies was 36.4 % (12/33).

### 3.7 Surgeries performed

Subtrochanteric Femoral De-Rotation Osteotomy (FDO) was the most common procedure reported in 6/7 studies^17^, ^18^, ^19^^,^^21^, ^22^, ^23^ and accounted for 90 out of 97 of all surgeries (92.8 %). Of the 6 studies reporting on FDO, one study by Lerch et al.^21^ reported on concomitant procedures during its 25 FDO surgeries. These included 24 surgical hip dislocations SHDs making up 96 % of 25 surgeries, 21 Cam Resections (84 %), 9 Labrum Fixation (36 %), 4 Acetabular rim trimming (16 %), 2 Acetabular cartilage treatment (8 %), 2 Femoral Cartilage treatment (8 %), and 1 Distalization of greater trochanter (4 %). Conversely, Tanaka et al.^20^ evaluated 7 cases of Transtrochanteric Rotational Osteotomy (TRO), making up 7.2 % of all cases reported in this review.

### 3.8 Pre-operative and post-operative impingement testing

#### 3.8.1 Preoperative

Table 2 includes the details of pre/postoperative impingement testing results. Preoperative impingement was reported in 6 of 7 studies.^17^, ^18^, ^19^^,^^21^, ^22^, ^23^ As an indication for surgery, general preoperative impingement was present in 100 % patients (n = 90) within the 6 studies. Anterior impingement was present in 85.1 % of patients (n = 47) reported in 4 studies. Posterior impingement was present in 100 % of patients (n = 39) in 2 studies. Unspecified impingement was present in 100 % of patients (n = 39) reported in 2 studies. Furthermore, Lerch et al.^21^ utilized preoperative FABER testing which was positive in 100 % of patients (n = 25) in the study.

**Table 2:** Follow-up Duration. Preop/postop for; measurements, Clinical tests/scores, Impingement, and Range of motion.

Study	Follow-Up	Pre-op. Impinge. Results	Post-op. Impinge. Results	Pre-operative Femoral Version in Degrees (S.D).	Other Angles/Values Measured:	Outcome Scores (S.D.)	Range-of-Motion Outcomes (S.D.)	Study	Follow-Up
**Cho (2021)**	2 years	+ Anterior Impingement	–	–	CCD: 145° (preop) 131° (postop) CEA: 42° (preop) 28° (postop)	Modified Harris Hip: 59 (preop) 85 (2yr postop) UCLA Activity: 4 (preop) 7 (2yr postop)	Flexion in neutral rotation: Preop 90°	Internal Rotation (IR) 90°/20° flexion: Preop 15°/25° Postop 30°/25°	External Rotation (ER) 90°/20° flexion: Preop 35°/65° Postop 45°/65°
**LErch (2022)****^23^**	1 year	**Increased FV with→** Anterior: 10/14 Posterior: 14/14 **Decreased FV with→** Anterior: 6/6	**Increased FV with →** Anterior: 0/14 *(p < 0.001) Posterior: 0/14 *(p < 0.001) **Decreased FV with →** Anterior: 1/6 *(p < 0.001)	**Increased FV**: 49**°** (±10**°**) **Decreased FV**: -5**°** (±4**°**)	**FPA (S.D.):** Inc. FV: 1.3**°** (±7**°**) Pre-op 4.5**°** (±6**°**) Postop* *(p=0.006) Dec. FV: 8.2**°** (±8**°**) Pre-op 0.5**°** (±5**°**) Postop* *(p=0.028)	**Merle d’Aubigne-Postel score (MdA)** **(18**–**0):** Inc. FV: 14 (1) preop, 17 (1) postop p<0.001* Dec. FV: 14 (1) preop, 17 (1) postop p<0.001* Subjective Hip Value (0–**100):** Inc. FV: 20 (22) preop 81 (11) postop p<0.001* Dec. FV: 23 (13) preop 72 (15) postop p<0.001* Modified Harris Hip: Inc. FV: 75 (11) postop Dec. FV: 77 (10) postop **HOOS Total**: Inc. FV: 72 (13) postop Dec. FV: 70 (8) postop **UCLA:** Inc. FV: 6 (2) postop Dec. FV: 5 (1) postop **WOMAC**: Inc. FV: 12 (9) postop Dec. FV: 13 (7) postop	**Flexion:** Inc. FV: Preop 103**°** (±12**°**) Postop 106**°** (±8**°**) (not significant) Dec. FV: Preop 94**°** (±7**°**) Postop 107**°** (±12**°**) (not significant) **Internal** **Rotation in 90° Flexion**: Inc. FV: Preop 48**°** (±12**°**) Postop* 28**°** (±8**°**) *p=0.003 Dec. FV: Preop 10**°** (±7**°**) Postop* 30**°** (±6**°**) p=0.028*	**ER In 90° Flexion**: Inc. FV: Preop 31**°** (±16**°**) Postop 38**°** (±9**°**) (not significant) Dec. FV: Preop 50**°** (22**°**) Postop 35**°** (8**°**) (not significant)	**IR in Extension:** Inc. FV: Preop 57**°** (±14**°**) Postop* 34**°** (±5**°**) *p=0.002 **External** **Rotation in Extension:** Inc. FV: Preop 17**°** (±9) Postop* 39**°** (±14**°**) *p=0.003
**Lerch (2022)****^21^**	4 years	Anterior: 22/25 Posterior: 25/25 FABER: 25/25	Anterior: 5/25 (p < 0.001)* Posterior: 1/25 (p < 0.001)* FABER: 3/25 (p < 0.001) * (Unreported whether isolated or concomitant. Therefore, total rate not calculable.)	46**°** (±9**°**)	**Acetabular Version (S.D)**: 21**°** (±5**°**) preop **Femoral version contralateral side (S.D.)**: 40**°** (±9**°**) preop	**Subjective Hip Value**: 24 preop, 84 postop p<0.001* Normalized WOMAC: 11 (8) postop **UCLA**: 6 (2) postop	**Flexion**: Pre-op 102**°** (±14**°**) Postop 108**°** (±10**°**) (not significant) **Extension**: Pre-op 5**°** (±5**°**) Post-op 8**°** (±7**°**) (not significant) **IR in 90** **Flexion:** Pre-op 48**°** (±13**°**) Postop* 34**°** (±18**°**) *p=0.017	**ER in 90** **Flexion**: 39**°** (±19**°**) preop 43**°** (±16**°**) postop (not significant) **Abduction in Extension**: Preop 38**°** (±8**°**) Post-op 37**°** (±10**°**) (not significant) **Adduction in Extension**: Preop 18**°** (8**°**) Postop 25**°** (±7**°**) (not significant)	**IR in Extension**: Preop 57**°** (±16**°**) Postop 37**°** (±17**°**) (not significant) **ER in Extension**: Preop 16**°** (±8**°**) Postop 44**°** (±16**°**) (not significant)
**Mastel (2020)****^18^**	12 months	29/29	7/29	−3.1**°** (−21**°** to 4**°**)	**CEA (Range):** 31.8**°** (18**°**-47**°**) preop	**iHOT-33: Avg.** Preop: 33 Post-op: 70.6 Improvement: 37.7* Range = (13–70) *p<0.001 **Patients Reporting subjective significant** **improvement**: 28/29	**Internal rotation: in degrees (range)** Pre-op 6 (0–15)/post-op 29.7 (15–45) difference: 23 *p<0.001 **External rotation: in degrees (range)** Pre-op 70.6 (45–90)/post-op 43.4 (30–70) difference: 27 *p<0.001		
**Mastel (2022)****^17^**	17.9 months	10/10	5/10	−0.5**°** (−17**°** to 5**°**)	**CEA (Range):** 33.5**°** (25**°**-47**°**) preop	**iHOT-33**: Avg. Preop: 33 Post-op: 77.3 Improvement: 37.7* Range = (14–58.8) *p<0.05 **Patients Reporting subjective significant** **improvement**: 10/10	**Internal rotation: in degrees (range)** Pre-op 5 (−5 to 10)/post-op 25 (20–50) difference: 22.5* (15–40) *p<0.05 **External rotation: in degrees (range)** Pre-op 70 (60–90)/Postop 50 (40–60) difference: 22.5* (−40 to −10) *p<0.05		
**Tamaki (2020)**	12 months	Anterior: Positive	Anterior: Negative	−7.1**°**	**CEA:** 23.2**°** (preop)	**JOA Score**: Pre-op: 77 Post-op: 99	**Internal Rotation**: Pre-op 10**°**/Post-op 52**°** **Flexion**: Pre-op 90**°**/Post-op 115**°**		
**Tanaka (2019)**	9.4 years	–	–	Ante/retroversion of femoral head as determined by location of OA. (Intact posterior articular surface = retroversion, and vice versa.)	**Yasunaga's** **Joint** **Congruency:** OA cohort before/after TRO: 2 Poor, 4 Fair/4 Fair, 2 Good ON cohort before/after TRO: Fair/Good	**MdA score (18**–**0):** **OA cohort**: (preop/postop) - Total 10.3/14.2 - pain 2.2/4 - mobility 5.2/5.7 - walking 3/4.5 **ON cohort**: Unable to report—reported mean included excluded surgeries.	–		

FV – Femoral Version; S.D.—Standard Deviation; IR – Internal Rotation; ER – External Rotation; CCD – Cleidocranial Dysplasia; CEA – Center Edge Angle; JOA—Japanese Orthopedic Association; FAI—Femoroacetabular Impingement; OA—Osteoarthritis; ON—Osteonecrosis; Post-op—Post operative; Preop—Pre-operative; WOMAC-- Western Ontario and McMaster Universities Osteoarthritis Index; FABER—Flexion, Abduction, External Rotation; HOOS—Hip Disability and Osteoarthritis Outcome Score; FPA—Foot Progression Angle; iHOT-33--International Hip Outcome Tool.

#### 3.8.2 Postoperative

Postoperative impingement was reported in 5 of 7 studies.^17^, ^18^, ^19^^,^^21^^,^^23^ Anterior impingement was reported in 13 % (n = 46) of patients in 3 studies (a patient from a case study was included in the preoperative impingement testing without subsequent postoperative results). The remaining data shows ∼72 % reduction in anterior impingement in these patients. Posterior impingement was reported in 2.6 % (n = 39) of patients in 2 studies. This represents a reduction in posterior impingement of 97.4 % among these patients. Unspecified impingement was reported in 30.8 % (n = 39) of patients in 2 studies. This represents a 69.2 % reduction in unspecified impingement in these patients. Lerch et al.^21^ found that postoperative FABER testing was positive in 12 % of patients (n = 25) in the study. This represents an 88 % postoperative reduction in positive FABER testing.

### 3.9 Preoperative measurement of femoral version (FV)

Table 2 includes the details of preoperative FV measurements as reported in 5 of 7 studies. Given Schmaranzer et al.’s^24^ establishment of normal version being 10–25° of anteversion—all studies reporting a mean with FV < 10° of anteversion were considered ‘retroverted’, and studies reporting a mean FV of >25° and were considered ‘anteverted’. Four studies^19^, ^20^, ^21^^,^^23^ comprising 46 patients report Retroverted FV with a weighted mean of −2.87°, and 2 studies^18^^,^^23^ comprising 39 patients report an Anteverted FV with a weighted mean of 47.08°.

### 3.10 Other angles/values measured

All 7 studies^17^, ^18^, ^19^, ^20^, ^21^, ^22^, ^23^ measured other angles (Table 2). Statistically significant post-operative improvement in Foot Progression Angle (FPA) was reported in both FAI patients with anteverted and retroverted FV.^23^ Likewise, Cho et al.^22^ commented that ‘FPA normalized’ postoperatively though provided no values. Tanaka et al.^20^ evaluated patients via Yasunaga's Joint Congruency Classification^25^ and found that 3/6 (50 %) of Osteoarthritis (OA) patients improved a measure of magnitude after TRO, while 1/1 osteonecrosis (ON) patient improved from ‘fair’ to ‘good’. Preoperative Central Edge Angle (CEA) was reported in 4 studies with a weighted mean of 32.25° (n = 41 hips).^17^^,^^18^^,^^20^^,^^22^ Cho et al.^22^ (n = 1) was the sole study to report postoperative CEA of 28°. Caput-Collum-Diaphysis Angle (CCD), FV of contralateral side, and Acetabular Version were also included in individual studies as reported in Table 2.

### 3.11 Outcome scores

All 7^17−23^ studies reported a variety of outcome scores. Both studies by Lerch et al.^21^^,^^23^ reported statistically significant postoperative improvements in Subjective Hip Value. Likewise, in the Lerch et al.^23^ study where patients were divided by preop anteverted or retroverted groups, the Merle d’Aubigne-Postel Score (MdA) significantly improved in both groups. MdA scores were also reported in OA cohort of Tanaka et al.’s^20^ study and showed postoperative improvement in each scored section, though no significance was reported. Both Mastel et al. studies^17^^,^^18^ showed significant postoperative improvement (37.7 points, n = 39) in the International Hip Outcome Tool (iHOT-33), and a ‘patient-reported subjective improvement’ in 38/39 patients (97 %). Among other outcome scores, only the UCLA Activity Score was reported in greater than 2 studies, where 3 studies^21^, ^22^, ^23^ reported postoperative scores with a weighted mean of 5.82 (n = 41 patients). However, Cho et al.^22^ was the only to report preoperative UCLA score of 4. The remaining outcome scores were not reported to be statistically significant or were in 2 or fewer studies, and thereby not indicated for aggregate analysis. Further details for each score from all 7 studies can be found in Table 2. Previously unmentioned outcome scores included: Modified Harris Hip Score, Western Ontario and McMaster Universities Osteoarthritis Index (WOMAC), and Hip Disability and Osteoarthritis Outcome Score (HOOS).

### 3.12 Range of motion outcomes

Range of motion measurements were reported in 6^17−19,21–23^ out of 7 studies and included preop/postoperative measurements are found in Table 2. Internal Rotation at 90-degree flexion (IR) was reported in 6 of 7 studies. Weighted means from 5 studies totaling 47 retroverted hips reported 6.57° of IR preoperatively, and 29.34° postoperatively (change in IR = +22.77°). Weighted means from 2 studies^18^^,^^23^ totaling 39 anteverted hips reported 48° of IR preoperatively, and 31.8° postoperatively (change in IR = −15.2°). Four^17^^,^^18^^,^^21^^,^^23^ out of 6 studies reported significant IR changes (Table 2). Likewise, external rotation at 90-degree flexion (ER) was reported in 5 of 7 studies and analyzed similarly. Weighted means from 46 retroverted hips reported 67° of preoperative ER, and 43.8° postoperative (change in ER = −23.2°). Weighted means from 39 anteverted hips reported 36.13° preop ER, and 41.2° postop (change in ER = +5.07°). 3^17,18,23^ of the 5 studies reported that their ER changes were significant. Beyond these calculations, heterogeneity in ROM reporting prevented further aggregation analysis of the data reported in Table 2. Lerch et al.^23^ reported the only other statistically significant postoperative improvement with a +22-degree increase in ‘ER in Extension’ in the anteverted hip cohort (n = 14). The remainder of ROM reporting is reported in Table 2.

### 3.13 Post-operative complications, reoperations and other outcomes/salient findings

#### 3.13.1 Complications

Postoperative complications were only reported in the 2 studies by Mastel et al.,^17^^,^^18^ totaling 39 patients (Table 2). 19 complications were reported among the 39 patients, though percentages and rates cannot be calculated as 1 case may have multiple complications. Given the isolated nature of the data coming from only 2 studies, aggregate analysis was not performed.

#### 3.13.2 Unplanned reoperations

Table 3 provides a full account of reoperations as reported in 4 of 7 studies^17^^,^^18^^,^^21^^,^^22^ (n = 65) with a 40 % incidence of reoperation (n = 26). Hardware removal was the most common reoperation, accounting for 77 % (n = 20) of all reoperations. Other surgeries/indications included: 1 anteverting periacetabular osteotomy, 2 to correct non-union, 1 insertion of femoral nail, and 2 for management of heterotrophic ossification. Other outcomes and salient findings are reported in Table 3.

**Table 3:** Complications, Reoperations, outcomes, and Salient Findings.

Study	Post-op Complications	Reoperations	Other Outcomes	Salient Study Findings
**Cho (2021)**	–	Fixation for osteotomy removed at 2yrs	Union of osteotomy site observed at 2yr Follow-up - Foot progression angle normalized	- Femoral varus De-Rotation osteotomy with surgical hip dislocation is a rational and appropriate option for definitive management in patients with extra-articular FAI, refractory to prior arthroscopic FAI surgery. - Extra-articular causes of FAI should be suspected in every refractory case.
**Larch (2022)**	–	–	**In-Toeing:** Inc. FV: 5 preop, 2 postop (**p=0.021**) Dec. FV: 0 preop, 1 postop **Out-Toeing**: Inc. FV: 0 preop, 0 postop Dec. FV: 2 preop, 0 postop	- Patients with increased FV that underwent FDO walked with less in-toeing postop. - Incr. FV patient with FDO had sig. increased FPA postop with no sig. difference compared to control group. - Subjective hip value of all patients increased significantly postop.
**Lerch (2022)**	–	**17/25 patients:** 16 Complete Hardware Removal (−3 with Hip Arthroscopy) 1 Anteverting PAO for severe acetabular retroversion.		- FDO is safe and effective for mainly female patients with high femoral version
**Mastel (2020)**	**14/29:** 3 un-displaced fractures involving osteotomy during surgery-- Cerclage wire used for correction. 3 delayed union 2 oligotrophic non-union 3 HO 2 trochanteric bursitis 1 post-op spinal headache	**4/29:** 2 for non-union 1 for insertion of fem. nail 1 for HO prominence pressure on sciatic nerve		–
**Mastel (2022)**	**5/10:** 2 HO 3 Hardware removal	**4/10:** 3 for hardware removal for non-union 1 for HO		- Significant improvement in patient reported and clinical findings with FDO. - This Author screens for FV in anyone w/impingement + IR < 15**°** on 90**°** flexion.
**Tamaki (2020)**	–	–		–
**Tanaka (2019)**	–	–	**Postop femoral head osteophyte formation**: OA: 5/6 (85 % patients) −50 % anterior location −17 % inferior location ON: 0/1 **Progressive joint space narrowing:** OA: 3/6 (50 % of patients) ON: 0/1 *1 Hip with widened/improved joint space over 4 years.	TRO enables repair and prevented progression of OA even in highly degenerative cases. Kellgren and Lawrence system for progression of OA evaluated postoperative outcomes: −4 hips graded at 2 −3 hips graded 3

ROM—Range of Motion; FAI—Femoroacetabular Impingement; FV—Femoral Version; FDO—Femoral Derotational Osteotomy; FPA—Foot Progression Angle; PAO—Periacetabular osteotomy; HO—Heterotopic ossification; IR—Internal Rotation; OA—osteoarthrosis; TRO-- Transtrochanteric Rotational osteotomy.

### 3.14 Methodological assessment and risk of bias

Using JBI Clinical appraisal tools for each study type: 2 case reports had a mean score of 6.5 out of 8. 2 case series had a mean score of 9.5 out of 10, and 3 retrospective cohort studies had a mean of 10.33 out of 11. Both reviewers scored each study the same, with no discrepancies. These scores warrant inclusion without unnecessary risk of bias.

## 4 Discussion

This systematic review evaluates the role of FDO for symptomatic FAI most commonly due to increased or decreased FV. The resultant findings highlight the role of impingement and ROM testing, and preoperative imaging measurements in indicating FDOs. This study reports significant improvements in clinical and ROM outcomes, further establishing FDOs as efficacious in reducing symptom burden and improving patients’ subjective evaluation of their condition. Of note, a similar SR was published concomitant to the writing of this study that likewise endorses the utility of FDO. However, this study reports on different data points and analyzes more recent studies that were not included in the previous SR.^26^ As such, this study serves as a necessary companion to that prior work.

### 4.1 Indications for FDO

The most common indications for FDO include pain and impingement, abnormalities in ROM testing, and abnormal FV on imaging. Other types of femoral dysplasia such as Coxa Valga, or Cam and Pincer deformities typically lie outside the therapeutic window of FDOs and necessitate different types of osteotomies or hip arthroscopies for correction.^10^^,^^24^^,^^27^ A full review of the indications and surgical management of various types of hip dysplasia is beyond the scope of this review. However, regarding the indications of FDO, this study aggregated preoperative FV data and found that retroverted femurs displayed a mean of −2.87° FV with preoperative measures of 6.57° of IR, and 67° ER. While anteverted femurs displayed a preoperative mean of 47.08° FV with preoperative measures of 48° of IR, and 36.13° ER. These clinical and radiological values provide literature with a step toward the establishment of unique clinical indications for FDO. This is useful as currently hip arthroscopy and FDO can both be used to treat some of the same clinical indications such as impingement, limited ROM, and femoral version abnormalities.^27^^,^^28^ Further clarification within these indications would reveal if or when it would be wise to pursue primary FDO in patients with FAI due to femoral dysplasia, in an attempt to reduce patient cost and morbidity.

Furthermore, due to its minimally invasive nature and low rates of complications and reoperation, hip arthroscopy is the first line surgical management for FAI. However, this study analyzes FDO patients being treated for refractory impingement after undergoing arthroscopy, and a study by Mastel et al.^17^ (underpowered-- so improved outcomes were not significant) also raises question of whether FDO may yield equal-to-slightly better clinical outcomes compared to arthroscopy. Taken together, this establishes a need to identify the patient populations and indications that may predict better outcomes with a primary FDO, as compared to arthroscopy. By identifying patients who are more likely to experience failed arthroscopic treatment, patients can avoid unnecessary surgeries, cost, and morbidity.^4^ Of note, this study also includes cases of hip arthroscopy concomitant with FDOs.^21^ This technique represents a viable option for primary management in dysplastic femurs with increased FV as well as other dysplastic features such as cam or pincer deformities identified by increased alpha or beta angles on imaging.

### 4.2 Outcomes scores, FV, and ROM

The data reporting outcomes scores within analyzed studies yielded a heterogeneous sample. Significant postoperative improvements were seen in multiple scoring systems including Subjective Hip Value, MdA score, iHOT-33, and in ‘patient reported subjective improvement’ which support conclusions of overall subjective clinical improvements in patients after undergoing FDO. These conclusions are limited by relatively small sample sizes, and some scores either not reporting significance or not having pre-operative scores to allow for comparative analysis (Table 2).

Additionally, this study is limited in its ability to comment on the degree to which FDO corrects the aberrant FV angles due to lack of reported post-operative FV measurements. However, efficacy can be inferred by the improved outcome scores discussed prior, as well as improved post-operative ROM (Table 2). After FDO, retroverted hips experience a 22.77-degree correction in IR, and anteverted hips with a 15.2-degree correction. Likewise, ER correction in retroverted hips reached 23.2°, and 5.07° in anteverted hips. This data is for reader information only as it is in the context of variable individual anatomy in that greater preoperative restriction of ROM likely leads to more postoperative correction. However, these significant changes in ROM do have clinical significance such as inducing improved gait patterns and less in-toeing.^23^ With post-operative ROM assessments and due to the young age in patients undergoing FDO, it seems appropriate to investigate return to sport (RTS) data after FDO. RTS data on distal femur rotational osteotomies, and high tibial osteotomies exist in the literature—though no studies seem to investigate RTS rates in patients with FAI treated by FDO. Such data could be useful in preoperative patient education and expectation management.

### 4.3 Rates of hardware removal

Reoperation rates after FDO were high where reported (40 %) with hardware removal accounting for majority, although indications for hardware removal (whether symptomatic or elective) were not reported. Saltzman et al.^29^ recently analyzed 7 years of ‘hardware removal’ procedures performed by recently trained orthopedic surgeons and found an overall complication rate of 9.6 %. This complication rate in hardware removal surgery provides insight into possible morbidity of FDO surgeries considering the high rates of hardware removal that follow. Future studies that report indications for hardware removal can distinguish the rates of planned and elective versus symptomatically indicated hardware removals. Such studies would provide further context for the reported outcomes of FDO, and potentially elucidate patient risk factors that could predict hardware removal.

### 4.4 Sparse complication data

Complications reporting in the studies analyzed was sparce. To this end, given the young patient population, future studies can evaluate long term outcomes and make promising contributions to the literature regarding the complications and benefits of these procedures. Likewise, studies exist that comment on how effectively various surgical interventions can delay the onset of OA,^30^ but more studies with high quality methodology and long-term follow-up are needed investigate the degree to which FDOs achieve this. Tanaka et al.^20^ touched on this with a postoperative assessment of OA progression using a Yasunaga score for each patient, though larger study samples would be of benefit to investigate FDOs utility more thoroughly in delaying OA onset.

### 4.5 Limitations

This study is limited by a small number of studies included for analysis (n = 7), as well as their levels of evidence (4 Level IV, and 3 Level III studies). Similarly, it is possible for studies to exist which possessed viable data for inclusion in this study, to evade earch due to title/abstract/keywords. Furthermore, the depth of analysis was limited by data being heterogenous or consisting of data points reported in too few studies, preventing summation or averaging. Additionally, 2 included studies were case studies, causing 5 studies to account for most results. A small number of physicians/institutions performed the surgeries, weakening the generalizability due to selection bias, and physician skill level and clinical preferences.

## 5 Conclusions

This study suggests that FDO is a relatively safe and reliable surgery in the definitive management of FAI due to aberrant FV. FDO leads to improvements in subjective evaluations, ROM, and clinical assessments of patients who underwent FDO for FAI. FDO carries a risk of reoperation for hardware removal, and further work with larger samples and prospective studies are needed to elucidate the nature and incidence of complications due to paucity of reporting in included studies. The strength of this study is in its exhaustive review and aggregation of data found in included studies that target the role of FDOs in the management of FAI due to increased FV. It is limited by small sample of studies, heterogeneous data, inherent limitations of reported data in studies, selection bias, small sample of surgeons, and possible existence of FDO data hidden in studies that were undetected by search methodology.

## Funding

This study did not receive funding.

## Ethical considerations

Informed Consent and IRB approval were not required as this is a non-human study.

## Ethical statement

The manuscript, “Femoral Rotational Osteotomies for Femoral Acetabular Impingement: A Systematic Review” did not require IRB approval as it analyzed the state of the literature and utilized data from peer-reviewed studies that reported on de-identified patient data.

## Funding statement

The research team for the manuscript titled “Femoral Rotational Osteotomies for Femoroacetabular Dysplasia: A Systematic Review” as submitted to the Journal of Orthopedics, collectively declares that there was no funding received to perform any part of this work.

## Patient consent statement

Regarding the manuscript “Femoral Rotational Osteotomies for femoroacetabular impingement: A systematic review”, *guardian and Patient consent was not needed* for this study as it was a review of studies including de-identified patients data.

## CRediT authorship contribution statement

**Chase T. Nelson:** Conceptualization, Data curation, Methodology, Writing – original draft. **Charles R. Reiter:** Data curation, Writing – review & editing. **Matthew Harris:** Table making. **Carl Edge:** Supervision, Writing – review & editing. **James Satalich:** Visualization, Supervision, Writing – review & editing. **Conor O'Neill:** Visualization, Supervision, Writing – review & editing. **John Cyrus:** Data curation, Methodology, Resources. **Alexander Vap:** Visualization, Conceptualization, Supervision, Project administration, Validation.

## Declaration of competing interest

The authors of this manuscript have no conflicts of interest to disclose.

## Figures and Tables

**Table:** 

1	((“Femoracetabular Impingement" [Mesh]) OR (“hip impingement" [Title/Abstract:∼3] OR “femur impingement" [Title/Abstract:∼3] OR “femoral impingement" [Title/Abstract:∼3] OR “Femoroacetabular impingement" [Title/Abstract:∼3] OR “Femoro-Acetabular impingement" [Title/Abstract:∼3] OR “femoroacetabular dysplasia" [Title/Abstract:∼3])) AND ((“Osteotomy" [Mesh] AND (rotation*[tiab] OR derotation*[tiab] OR de-rotation*[tiab] OR rotation*[ot] OR derotation*[ot] OR de-rotation*[ot])) OR (“rotation osteotomy" [Title/Abstract:∼3] OR “rotation osteotomies" [Title/Abstract:∼3] OR “rotational osteotomy" [Title/Abstract:∼3] OR “rotational osteotomies" [Title/Abstract:∼3] OR “derotation osteotomy" [Title/Abstract:∼3] OR “derotation osteotomies" [Title/Abstract:∼3] OR “derotational osteotomy" [Title/Abstract:∼3] OR “derotational osteotomies" [Title/Abstract:∼3] OR “de-rotational osteotomy" [Title/Abstract:∼3] OR “de-rotational osteotomies" [Title/Abstract:∼3] OR “de-rotation osteotomy" [Title/Abstract:∼3] OR “de-rotation osteotomies" [Title/Abstract:∼3]))	88

**Table:** 

1	exp osteotomy/and (rotation* or derotation* or de-rotation*).ti,ab,kw.	3497
2	((rotation* or derotation* or de-rotation*) adj3 osteotom*).ti,ab,kw.	1757
3	2 or 3	3982
4	exp femoroacetabular impingement/or (FAI or hip impingement or dysplastic hip impingement or femoroacetabular dysplasia).ti,ab,kw.	7049
5	((femur or femor* or Femoroacetabular or Femoro-Acetabular) adj3 impingement).ti,ab,kw.	4488
6	4 or 5	7629
7	3 and 6	102

**Table:** 

ID	Search	Hits
#1	MeSH descriptor: [Osteotomy] explode all trees	1026
#2	rotation* or derotation* or de-rotation*	9404
#3	#1 AND #2	51
#4	((rotation* or derotation* or de-rotation*) NEAR/3 osteotomy*)	53
#5	#3 OR #4	88
#6	MeSH descriptor: [Femoracetabular Impingement] explode all trees	113
#7	FAI or hip impingement or dysplastic hip impingement or femoroacetabular dysplasia	731
#8	((femur or femor* or Femoroacetabular or Femoro-Acetabular) NEAR/3 impingement)	223
#9	#6 OR #7 OR #8	736
#10	#5 AND #9	0
